# Iodide of Iron

**Published:** 1855-04

**Authors:** Richard W. Nelson

**Affiliations:** Buffalo


### BUFFALO MEDICAL JOURNAL

AND

### MONTHLY REVIEW.

VOL. 10. APRIL, 1855. NO. 11.

### ORIGINAL COMMUNICATIONS.

ART. I.—*Iodide of Iron.* By RICHARD W. NELSON, M.D. A paper read before the Buffalo Medical Association at its meeting for February, 1855.

Iodide of Iron was first used in the practice of medicine by the late Dr. Anthony Todd Thompson. It has been found very useful in diseases of debility, more especially in tuberculosis and strumous enlargement of the glands: being composed of one part of iodine, and one of iron, with five of water, it partakes equally of the properties of the ferruginous tonic, as of the absorbent and alterative power of iodine, and therefore can be employed in anœmia, chlorosis, and amennorrhœa with much advantage.

In consequence of its great deliquescence in substance, and its decomposing so rapidly in solution, many methods have been tried to preserve it unchanged, and avoid the irritation which so frequently happens when taken internally. The principal and most useful are the saccharated iodide of iron, proto-iodide of iron pill, the syrup of the iodide of iron, and the bin-iodide of iron fluid. I have been in the habit of using these different preparations myself for some years, and can, therefore, speak with confidence to their certainty of effect.

The saccharated iodide of iron is generally in small crystalline masses almost like the scales of the ammonio-citrate of iron, of an iron-gray color, opaque, and having a metallic lustre; it is perfectly neutral and soluble, and possesses the peculiar advantage of remaining so; the dose is from 2 to 5 grains. In this preparation we have the privilege of being able to combine it in the form of pill with any thing else that it may be necessary to give the patient. I have been mostly in the habit of ordering it in mass with the fel bovinum inspissatum, especially where the liver is inactive, or in that morbid irritability of the stomach accompanied with vomiting soon after meals, not depending on organic disease; there is a powder of a pale yellow color, called saccharated iodide of iron, made by adding saccharum lactis to the iodide of iron, the directions for making which are found in Grey's Ph. I would wish to caution the profession against its use, being almost inert and of but little value.

The next preparation is the proto-iodide of iron pills; Professor Christison, in Pharm. Trans., Aug. 1st, 1844, recommends a form communicated to him by Mr. Robert Leslie, late Apothecary in the Royal Infirmary of Glasgow, but the proto-iodide that I wish to draw your attention to more particularly, is that intruduced by Dr. Dupasquier, Physician to the Hotel Dieu, in the cure of phthisis. He says, "it is a remedy infinitely more useful than all those put together which have been employed to the present hour in that disease." "The proto-iodide of iron generally used," says Dr. Dupasquier, "is a medicine totally different both in its chemical nature and its therapeutic action, from the proto-iodide which I use: the former is not a proto-iodide, although so named in all the formulæ, but a mixture; the composition varies according to the greater or less precautions employed in its preparation and conservation. When dissolved in water, the liquid, instead of being colorless, or scarcely colored green, is brown, more or less deep, according as the iodide has been more or less exposed to the air. The smell and taste indicate the presence of a notable quantity of free iodine. A properly prepared solution should have no smell, nor more taste than the other salts of iron. It should have no apparent action on amidine, and with yellow cyanide of potassium it should afford a white precipitate; but with both of these reägents the solution of common proto-iodide of iron affords a blue powder; in the latter case a very deep blue."

By these observations we perceive that the iodide of iron of commerce differs most materially from the true proto-iodide of iron, which, properly administered in that awful bane of the human family, pulmonary consumption, tends to most favorable results. "Clinical observation has convinced me," continues Dr. Dupasquier, "that the least alterative in the colorless solution of the proto-iodide is sufficient to diminish its medical properties, and to communicate to it an irritating action, which totally changes the effects of it." Is it any wonder then, that the iodide of iron should so often fail, seeing it is so frequently exhibited in the irritating decomposing state, instead of the true proto-iodide of iron?

The following is the formula for the pilulæ proto-iodide Ferri:

| | | |
|---|---|---|
| Take of Iodine, | - - - - | 121 grains. |
| " Iron, | - - - - - - | 242 " |
| " Distilled water, | - - - | 378 " |

Introduce the whole into a small matrass, which hold plunged during eight or ten minutes in water heated to about 170° F., so that no portion of the iodine shall be volatilized. Agitate the mixture frequently. At first the liquid becomes brown, but soon becomes perfectly colorless, or at most retains a nearly imperceptible green hue. This preparation must be extemporaneous, for it would be a vain attempt to preserve it unaltered for one hour, even in ground-stopple-bottles and although metallic iron were present, owing to the decomposition of the water; filter, and pour the solution into an untinned iron vessel; add Narbonne honey, 302 grains; evaporate rapidly, until a great part of the original water be dissipated, and a syrupy consistence shall be attained; then add at intervals, continually agitating with an iron spatula, powder of gum tragacanth, 184 grains: form a mass, and divide into 200 pills. Each pill will contain almost exactly three-quarters of a grain of proto-iodide of iron. These pills will remain a long time unaltered.

I shall say but little on the syrup of the iodide of iron: it is in a form on which I have little dependence. Some may consider it useful, but it so soon spoils, the best preparation not keeping longer than a month, that I deem it uncertain, and never prescribe it now, especially having three other valuable forms; however, as some may still wish to exhibit it, I give Dr. Dupasquier's recipe for making it:

| | | |
|---|---|---|
| Take of Iodine, | - - - - | 15.12 grains. |
| " Iron filings, | - - - | 30.23 " |
| " Distilled water, | - - - | 120.89 " |

Proceed with these to form a normal solution as for the pills:

Take of the normal solution, 60½ Troy grains,
Syrup of gum Arabic, colorless and thick, 6.3 Troy ounce weight,
Syrup of orange flowers, 1.575 Troy ounce weight.

Mix perfectly by agitation during a few moments. It is indispensable that the syrups be colorless, so that the physician may have ocular proof that the medicine is not altered or injured. They should have more than usual consistence in order that the addition of the normal solution shall not impart such fluidity as would facilitate the alteration of the ferruginous salt by contact of air.

The proto-iodide is preserved in a state of perfection, in this formula, for at least a month.

The total quantity of the above syrup is eight ounces by weight, the total quantity of the proto-iodide is eight grains, that is, one grain to every ounce weight of syrup: every tablespoonful will, therefore contain two-thirds of a grain of proto-iodide of iron.

The next and last preparation we come to is the Liquor Ferri Bin-iodide: this was introduced to the profession twelve or thirteen years ago by Dr. Wm. Tyson, Derbyshire, England. It is very necessary for us to have different preparations of the same medicine, as we frequently meet with patients who strongly object to swallowing pills, and sometimes vice versa fluids, and we are thus able to cater to their tastes in this very valuable article of medicine; it is also a form which can be very easily exhibited to young children.

℞ Potassæ Hydriodat, ʒss.

Aquæ puræ, ʒx.; misce, et adde,

Liq. Ferri oxysulphatis, ʒij. M.

Dose gutt. xx–xxx bis die.

This solution is of a beautifully deep red color, and transparent. It contains, like most other iodides, a little free iodine, but retains its color, and does not part with its iron. It will keep for years. The liquor oxysulphatis ferri is also a test for the purity of hydriodate of potash, producing in the above proportions a deep transparent solution.

The following is the formula for preparing the liq. ferri oxysulphatis:

℞ Ferri Sulphatis, ʒii, (or ʒiij).

Acidi Nitrici, ʒiij.

Aquæ, ℥iss.

Mix the nitrate acid carefully with the sulphate of iron for one quarter of an hour, then by degrees add the water; finally strain through paper.

It now remains for me to speak of the mode of administration of the iodide of iron.

It is particularly necessary to caution patients who are using any of the preparations of iodine, against the indulgence of every sort of food containing sugar or starch, as they decompose the preparations of iodine, converting them into the ioduret of amidine, which can be easily traced in the stools of those who eat bread, potatoes, rice, gruel, and vegetables, while taking the medicine. Dr. Majsisovitz, of Vienna, was the first, I believe, who directed the attention of the profession to this fact. I generally place my patient on a solid meat diet, with the use of but very little bread or baked potatoes, and sign the pill, or drops, to be taken immediately after meals; by this precaution we avoid the irritating consequence so likely to happen to patients taking the iodide of iron on an empty stomach.

The peculiarly characteristic constitutional symptoms, known as the crisis, which frequently appear under the exhibition of iodine, are: first, inflammation of the Schneiderian membrane, causing a sensation like cold in the head, with increased discharge of mucus; this is particularly noticed in the exhibition of hydr. pot. Secondly, irritability of temper peculiarly noticed by myself. Thirdly, a cutaneous eruption, resembling miliaria, and even papular, sometimes pustular, first appearing on the face, then the throat, and extending afterward over the surface of the body; I never saw the pustular form of eruption from the use of any preparation of iodine, but the hydriod. potassæ. Lastly, salivation; the salivation from iodine differs essentially from mercury, by not producing ulceration of the gums or fœtor of the breath, and rapidly subsiding on the cessation of the medicine.

When these symptoms appear, even before salivation, I consider it necessary to withdraw the medicine for a time, from one to three weeks, during which rest the beneficial effects of the compounds of iodine are most likely to develop themselves, and before which time I rarely look for them.

BUFFALO, January 26, 1855.

